# Emotion Regulation Moderates the Association between Empathy and Prosocial Behavior

**DOI:** 10.1371/journal.pone.0096555

**Published:** 2014-05-08

**Authors:** Patricia L. Lockwood, Ana Seara-Cardoso, Essi Viding

**Affiliations:** Division of Psychology and Language Sciences, University College London, London, United Kingdom; University of Maribor, Slovenia

## Abstract

Theory and evidence suggest that empathy is an important motivating factor for prosocial behaviour and that emotion regulation, i.e. the capacity to exert control over an emotional response, may moderate the degree to which empathy is associated with prosocial behaviour. However, studies to date have not simultaneously explored the associations between different empathic processes and prosocial behaviour, nor whether different types of emotion regulation strategies (e.g. cognitive reappraisal and expressive suppression) moderate associations between empathy and prosocial behaviour. One hundred–and-ten healthy adults completed questionnaire measures of empathy, emotion regulation and prosocial tendencies. In this sample, both affective and cognitive empathy predicted self-reported prosocial tendencies. In addition, cognitive reappraisal moderated the association between affective empathy and prosocial tendencies. Specifically, there was a significant positive association between empathy and prosocial tendencies for individuals with a low or average tendency to reappraise but not for those with a high tendency to reappraise. Our findings suggest that, in general, empathy is positively associated with prosocial behaviour. However, this association is not significant for individuals with a high tendency for cognitive reappraisal.

## Introduction

Humans have a remarkable capacity to engage in prosocial behaviours, i.e. social behaviour intended to benefit another, with genetically unrelated individuals [1]. However, the processes that influence how and when prosocial behaviours occur remain poorly understood. Theory and evidence suggest that empathy, i.e. the capacity to understand and/or resonate with the affective experiences of others [2], is one of the key motivating factors for prosocial behaviour [3]–[5].

A number of processes are thought to contribute to the experience of empathy. These include ‘affective’ empathic processes, such as being aware of and resonating with the feelings of another individual, as well as ‘cognitive’ empathic processes, such as identifying and understanding what another individual is thinking or feeling without a necessary affective response [1]. There is evidence that processes related to affective and cognitive empathy are positively associated with prosocial behaviour (for a review see [3]). The majority of these studies have used the interpersonal reactivity index (IRI, [6]), which measures dispositional empathic concern/sympathy, or cardiovascular and electrodermal indices, such as heart rate deceleration and facial electromyographic (EMG), as proxy measures of affective empathy. For example, heart rate deceleration (which is thought to index vicariously induced sadness or sympathy, e.g [7]) and increased indicators of facial sadness when watching needy others are associated with increased willingness to help [8]. Dispositional empathic concern, as measured by the IRI, has also been linked to higher levels of self-reported charitable giving [9] and greater self-reported concern for the welfare of others [10]. In terms of associations between cognitive components of empathy and prosocial behaviour, studies have focused on correlating the perspective-taking subscale of the IRI to self-reported prosocial behaviour and have found that trait perspective taking is positively associated with frequency of volunteering [11] and self-reported prosocial tendencies [12]. It should be noted, however, that the empathic concern and perspective taking scales of the IRI tap constructs that, although related, are different from the current conceptualisation of ‘affective’ and ‘cognitive empathy’ [2]. Nonetheless, together, these studies broadly suggest that affective and cognitive empathic processes may motivate prosocial behaviour.

Whilst it is often assumed that an empathic response to another’s distress will motivate prosocial behaviour, Eisenberg [13] points out that association between the two constructs are often modest and sometimes weak. A possible reason for these modest associations is the influence of moderating variables [13]. It has been suggested that emotion regulation, i.e. the capacity to modulate or exert control over an emotional response, might be one such moderator variable [14], [15]. Eisenberg and Fabes [14] propose a model whereby individual differences in both the emotional intensity and regulation capacities are related to an individual’s level of prosocial responding. Specifically, they suggest that the perception of distress in another leads to emotional arousal, but emotion regulation i.e. and how this arousal is evaluated by the observer, will influence the subsequent goal directed behaviour, either to improve their own situation or help the others’ situation [14]. The degree of emotion regulation during a state of emotional arousal (over-, optimal-, or under-regulation) is also proposed to relate to the likelihood of prosocial behaviour. For example, individuals who are able to optimally regulate their arousal, so that they do not experience undue distress in the face of another person’s emotions and thus do not become self-focused, are proposed to behave prosocially [14]. In contrast, individuals who are over- regulated are proposed to exhibit proactive withdrawal, which inhibits prosocial behaviour. Finally, those who are under-regulated are proposed to be prone to aggression and thus more likely to exhibit antisocial rather than prosocial behaviour in an emotionally arousing situation [14].

The model outlined by Eisenberg and Fabes [14] discusses the degree of emotion regulation (over-, optimal-, or under-regulation) as important for linking empathy to prosocial behaviour. However, it is also likely that the type of emotion regulation strategy used will be important. Both cognitive reappraisal and expressive suppression represent emotion regulation strategies [16]–[18]. Cognitive reappraisal involves reinterpreting an emotional response so that the intensity of its emotional impact is modified [19]. For example, re-framing a distressing situation as a situation where someone will benefit from support, as opposed to a situation where someone is emotionally labile and potentially unpleasant. Consequently, cognitive reappraisal will enable a person to focus on strategies to provide constructive helping behaviours, rather than the aversive qualities of the situation. Cognitive reappraisal is thought to be a successful emotion regulation strategy, decreasing negative affect and resulting in an attenuation of blood pressure [20], [21]. In contrast, expressive suppression involves actively inhibiting on-going emotion-expressive behaviour [17], [18], [22]. For example, managing an emotional response to an aversive situation in an effortful manner such that cognitive resources are consumed. Expressive suppression is thought to be a suboptimal strategy because it creates a conflict between heightened emotional arousal and overt expression of the arousal [17], [18], [23]. These two types of emotion regulation strategies also appear to lead to different outcomes and consequences for interpersonal functioning [16], [24]–[26]. Whilst cognitive reappraisal is positively related to having closer relationships with friends, fewer depressive symptoms and greater life satisfaction, expressive suppression is associated with greater experience of negative emotions, disturbed interpersonal interactions, avoidance of close relationships and reports of less life satisfaction and optimism [16], [24]–[26].

Despite evidence linking empathy to prosocial behaviour (e.g. [8], [11]) and the proposal that individual differences in emotion regulation may moderate associations between empathy and prosocial behaviour [14], [15], this has not, to our knowledge, been directly examined. Moreover, how distinct emotion regulation strategies might moderate associations between empathy and prosocial behaviour has not been explored. The majority of studies suggesting empathy as a motivating factor for prosocial behaviour have investigated self-reported empathic concern (feeling ‘for’ another person, including compassion and sympathy, e.g. [9], [10]), rather than self-reported affective empathic responses (the ability to vicariously experience the emotional experience of others; or feeling ‘as’ another individual). While these two processes are no doubt closely related, there is a lack of empirical data regarding how feeling in a similar emotional state to another may motivate prosocial behaviour. In addition, self-reported cognitive empathic ability (i.e. the ability to position oneself ‘in another person’s shoes’) might also relate to prosocial behaviour, but compared to the role of affective empathic processes motivating empathy this has received relatively little attention to date (c.f. [11], [12]).

On the basis of previous research and theory (e.g. [3], [10], [12]), we predicted that both dispositional cognitive and affective components of empathy would be associated with increased prosocial tendencies, but the amount of variance in prosocial behaviour explained by the two types of empathy may be unequal. We also tested interactions between the components of empathy (affective and cognitive) and types of emotion regulation strategy (cognitive reappraisal and expressive suppression) to examine whether individual differences in emotion regulation strategy moderate associations between empathy and prosocial behaviour.

## Methods

### Participants

One-hundred-and-ten healthy adults (50% males; 50% females) aged 18–33 (M = 21.9, SD = 3.7) were recruited through university participant databases (comprised of undergraduate and postgraduate students as well as non-student community members) and through online advertisement. Exclusion criteria included previous or current neurological or psychiatric disorder (as reported by the participants) and non-normal or non-corrected to normal vision. Participants were compensated at a rate of £8 per hour.

### Ethics Statement

All participants provided written informed consent and the study was approved by the University College London Clinical, Educational and Health Psychology Research Ethics committee.

### Procedure

Participants completed questionnaires to assess empathy, emotion regulation and prosocial tendencies as part of a larger battery of tasks and questionnaires.

### Questionnaires

#### Questionnaire of Cognitive and Affective Empathy (QCAE; [27])

The QCAE, is a multidimensional empathy questionnaire devised to measure the ability to comprehend the emotions of another (cognitive empathy) as well as the ability to vicariously experience the emotional experience of others (affective empathy). In the development of the QCAE, two raters selected items from other well-validated and commonly used empathy measures (e.g. Empathy Quotient; [28], Hogan Empathy Scale; [29], the Empathy subscale of the Impulsiveness-Venturesomeness-Empathy Inventory; [30], and the IRI; [9]) if they were deemed to measure affective or cognitive empathy. Items from these scales deemed to measure other processes (e.g. sympathy) were not included. A Principal Component Analysis of the selected items revealed five components (or sub-scales), further organized in two dimensions assessing cognitive and affective empathy. The cognitive empathy dimension comprises subscales measuring perspective-taking (e.g. “I am good at predicting how someone will feel”) and Online simulation (e.g. “Before criticizing somebody, I try to imagine how I would feel if I was in their place.”). The affective subscales assess emotion contagion (e.g. “People I am with have a strong influence on my mood”); peripheral responsivity (e.g. “I usually stay emotionally detached when watching a film”); and proximal responsivity (e.g. “I often get emotionally involved with my friends’ problems”). Items are rated on a 4-point scale from “strongly disagree” to “strongly agree”. The QCAE has good validity and internal consistency [27]. In the present study Cronbach’s alpha for cognitive empathy subscale .87; affective empathy subscale .88).

#### Emotion Regulation Questionnaire (ERQ; [19])

The ERQ is comprised of two dimensions that assess either reappraisal or suppression regulation strategies. The reappraisal dimension contains items such as “I control my emotions by changing the way I think about the situation I’m in” and the suppression dimension has items such as “I control my emotions by not expressing them”. Items are rated on a 7-point scale from “Strongly disagree” to “Strongly agree”. The ERQ has good construct validity and internal consistency ([19]; in the present study Cronbach’s alpha for reappraisal subscale. 73; suppression subscale. 87).

#### Prosocial Tendencies Measure (PTM; [31])

The PTM is a 23-item self-report measure that assesses various prosocial tendencies such as compliant prosocial tendencies (e.g. “When people ask me to help them, I don’t hesitate”), dire prosocial tendencies (e.g. “I tend to help people who hurt themselves badly”) and emotional prosocial tendencies (e.g. “I tend to help others particularly when they are emotionally distressed”). Items are rated on a 5-point scale from “Does not describe me at all” to “Describes me greatly”. The PTM has good construct validity and internal consistency ([31]; in the present study Cronbach’s alpha .86).

### Data Analyses

Bivariate correlations were corrected for multiple comparisons using Benjamini & Hochberg False Discovery Rate [32]. Corrected p-values are reported. Steiger’s Z tests (two-tailed) were conducted to test if the different types of empathy (i.e. affective and cognitive empathy) and the different types of emotion regulation strategies (i.e. cognitive reappraisal and expressive suppression) presented differential correlation coefficients with prosocial tendencies.

Moderation analyses were then conducted to investigate whether the affective or cognitive empathy subscales interacted with either types of emotion regulation (reappraisal or suppression) to predict prosocial tendencies. All predictor variables were mean centred prior to analyses. Separate regression models using either the affective empathy subscale of the QCAE (QCAE-affective empathy) or the cognitive empathy subscale of the QCAE (QCAE-cognitive empathy) at the first stage; the reappraisal subscale of the ERQ (ERQ-reappraisal) or the suppression subscale of the ERQ (ERQ-suppression) at the second stage; the interaction term between these variables at the third stage were run. Consequently, four regression models were examined. Interaction effects were tested in SPSS using PROCESS [33]. Significant interactions were followed up by examining the conditional effect of empathy on prosocial tendencies at 1 standard deviation (SD) below the mean, at the mean, and 1 SD above the mean of emotion regulation.

## Results

Bivariate correlations between questionnaire measures of empathy, emotion regulation and prosocial behaviour were examined (see Table 1 for a full list of correlations). QCAE-affective empathy and QCAE-cognitive empathy were both positively associated with prosocial tendencies (r = .36, p<.001 & r = .43, p<.001 respectively) and these correlations were not significantly different (z = −.80, p>.05). ERQ-reappraisal was not significantly correlated with prosocial tendencies (r = .11, p = .30). ERQ-suppression was significantly negatively correlated with prosocial tendencies (r = −.27, p = .006). These two correlations were significantly different (Z = 2.69, p<.05).

**Table 1:** Correlations between questionnaire measures.

	QCAE: CE	QCAE: AE	PTM total	ERQ: reappraisal
QCAE: AE	.417**			
PTM total	.433**	.358**		
ERQ: reappraisal	.333**	.173	.113	
ERQ: suppression	−.360**	−.529**	−.266**	−.089

Abbreviations: QCAE-AE, Questionnaire of Cognitive and Affective Empathy Affective Empathy subscale; QCAE-CE, Questionnaire of Cognitive and Affective Empathy Cognitive Empathy subscale; ERQ, Emotion Regulation Questionnaire; PTM, Prosocial Tendencies Measure.

*p<.05.

**p<.01.

To examine whether the associations between affective and cognitive empathy and prosocial behaviour were explained by joint variance between the two components or whether they uniquely predicted prosocial tendencies we ran an additional multiple regression analysis. There were unique associations between each empathy component and prosocial tendencies (affective empathy, t = 2.29, p = .024; cognitive empathy, t = 3.67, p<.001).

**Figure 1:**
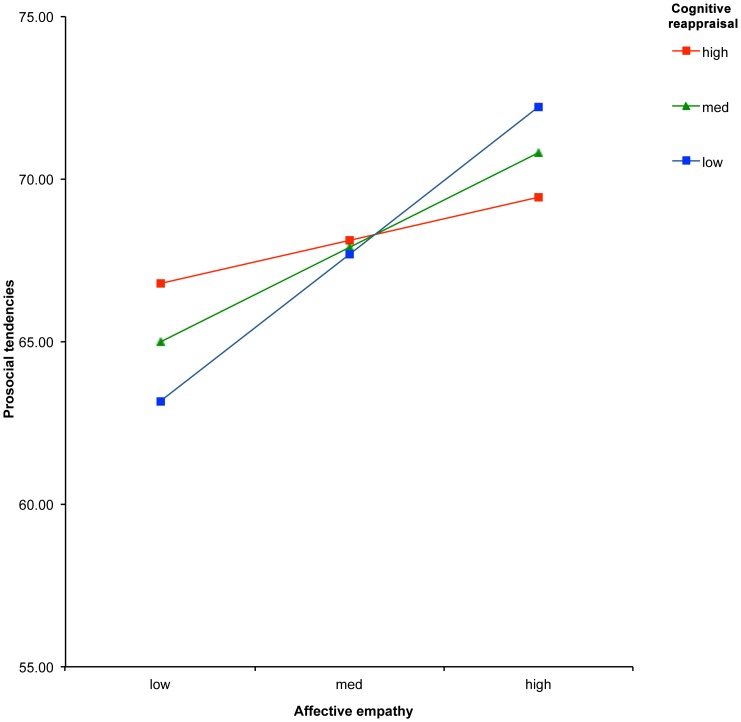
Moderation of the association between affective empathy and prosocial tendencies by cognitive reappraisal.

For the first regression model we entered QCAE-affective empathy (first stage), ERQ-reappraisal (second stage), and their interaction term [QCAE-affective empathy×ERQ-reappraisal] (third stage) as predictors of prosocial tendencies. This analysis revealed a significant positive association between QCAE-affective empathy and prosocial tendencies (t = 3.98, p<.001) but not between reappraisal and prosocial tendencies (t = .57, p = .570). Interestingly, the interaction between QCAE-affective empathy and ERQ-reappraisal was significant (t = −2.39, p = .019). At 1 SD below the mean on ERQ-reappraisal there was a significant positive association between QCAE-affective empathy and prosocial tendencies (t = 4.56, p<.001). There was also a significant association at the mean (t = 3.27, p = .002). However at 1 SD above the mean on ERQ-reappraisal the association between QCAE-affective empathy and prosocial tendencies was non-significant (t = 1.08, p = .282) (see Figure 1). In other words, affective empathy was associated with prosocial behaviour for those with low and average levels of cognitive reappraisal (with the steepest slope for individuals with lowest level of cognitive appraisal), but those with high levels of cognitive reappraisal presented similar levels of prosocial behaviour regardless of level of affective empathy.

For the second regression model, QCAE-cognitive empathy, ERQ-reappraisal and their interaction term were entered as predictors of prosocial tendencies. This analysis showed a significant positive association between QCAE-cognitive empathy and prosocial tendencies (t = 5.00, p<.001) but not between reappraisal and prosocial tendencies (t = −.39, p = .699). The interaction between QCAE-cognitive empathy and ERQ-reappraisal was not significant (t = −1.18, p = .243). This pattern of findings suggests that QCAE-cognitive empathy was positively associated with prosocial tendencies regardless of level of reappraisal emotional regulation strategies.

We also examined the interaction between the two QCAE subscales and ERQ-suppression and their association with prosocial tendencies. These two regression models showed that both QCAE-AE and QCAE-CE were positively associated with prosocial tendencies (t = 3.98, p<.001 and t = 5.00, p<.001) but ERQ-suppression was not significantly associated with prosocial tendencies in either model (t = −1.00, p = .32 and t = −1.36, p = .18). Neither of the interactions between QCAE-affective empathy or QCAE-cognitive empathy and ERQ-suppression were significant (both ps>.05).

## Discussion

The present study investigated associations between empathy and prosocial behaviour, and whether different types of emotion regulation strategy moderate associations between empathy and prosocial behaviour. We found that both affective and cognitive components of empathy were positively and uniquely associated with self-reported prosocial behaviour. Cognitive reappraisal, but not expressive suppression, played a role in moderating the association between empathy and prosocial behaviour. Specifically, level of cognitive reappraisal moderated the relationship between affective empathy and prosocial behaviour.

The finding that both affective and cognitive empathy are associated with prosocial behaviour supports previous studies suggesting that empathy is a key motivating factor for prosocial behaviour (e.g. [3], [8] [10], [12], [15]). Interestingly, associations between affective and cognitive empathy and prosocial behaviour were not significantly different. Additional analyses showed that cognitive and affective empathy uniquely predicted prosocial behaviour, suggesting that both empathy components play a role in motivating prosocial behaviour. Consequently, whilst it is likely that these two components will often work together in everyday life as they are moderately correlated (e.g. [27], [34]), our finding raises the possibility that having high levels of just one component could motivate prosocial behaviour, but this needs to be investigated further.

We also observed that expressive suppression was negatively associated with prosocial tendencies. This pattern fits with previous studies suggesting that expressive suppression is a maladaptive emotion regulation strategy [16], [24]–[26]. Our results extend these findings by suggesting that in, addition to being related to greater experience of negative emotions, avoidance of close relationships and reports of less life satisfaction [16], [24]–[26], expressive suppression is also associated with less self-reported prosocial tendencies.

The type of emotion regulation strategy was important for moderating associations between empathy and prosocial tendencies; cognitive reappraisal moderated associations between affective empathy and prosocial behaviour whilst expression suppression did not. In addition, the degree of emotion regulation interacted with the degree of empathy to predict prosocial behaviour. Affective empathy was positively associated with prosocial behaviour for participants at low and average levels of cognitive reappraisal. This positive association was not evident in participants who reported a high tendency to reappraise. Instead, these individuals had similar levels of prosocial tendencies regardless of level of affective empathy.

Consequently, although empathy is generally assumed to have a significant positive association with prosocial behaviour [3], [4] this may not be the case for all aspects of empathic processing. Our finding suggests that affective empathy is an important motivating factor for prosocial behaviour only for particular individuals, which fits with accounts considering a multitude of factors involved in motivating prosocial behaviour [5]. One explanation is that those with high tendency to reappraise are (at least according to their self-report) more able to change their strategy and viewpoint when evaluating the situation at hand. This capacity may allow one to more readily deduce the desirability of prosocial behaviour even without the experience of the affective components empathy. Whilst we observed a significant moderation of cognitive reappraisal on the association between affective empathy and prosocial behaviour, moderation effects were not evident for associations between cognitive empathy and prosocial behaviour. This lack of association could be because of the overlap in processes involved in cognitive empathy and those involved in cognitive reappraisal. Indeed self-reports of cognitive empathy and cognitive reappraisal were positively correlated in this sample. Processes such as shifting perspective or attention are common to both cognitive empathy and reappraisal. In terms of increasing prosocial behaviour in those individuals high in reappraisal, it is possible that promoting cognitive empathy might elevate the motivation of these individuals to behave prosocially.

Interestingly, we also found that those with the highest levels of self-reported prosocial behaviour were individuals low in reappraisal but high in affective empathy. Given that cognitive reappraisal is positively related to interpersonal functioning [16], [24]–[26] and prosocial behaviour is generally seen as a positive aspect of interpersonal functioning this result may seem somewhat surprising. In addition, the model proposed by Eisenberg & Fabes [14] suggests that those high in experiences of emotional intensity and low in emotion regulation would not manage appropriate prosocial responding and might even display antisocial/aggressive behaviour in response to emotional arousal. However, it has been suggested that high levels of prosocial and altruistic behaviour are not always beneficial and there are cases when acts that are subjectively prosocial can be, to the observer, objectively unhelpful [35]. Future research needs to determine whether the self-reported prosocial behaviours by individuals with high affective empathy and low cognitive appraisal capacities are perceived as objectively helpful/prosocial by the observer. Items on the prosocial tendencies questionnaire assess the self-reported tendency to engage in prosocial behaviours, rather than the quality of them. Future studies could include experimental and/or observational measures to examine this. The types of prosocial responses of individuals high in affective empathy and low in cognitive reappraisal could be compared to those high in cognitive reappraisal and high in affective empathy. Another promising avenue for future research is to investigate empathy components and emotion regulation strategies in tandem in clinical populations thought to be characterised by atypical empathy and emotion regulation. For example, autism spectrum disorders, psychopathy and alexithymia have all been associated with both atypical empathy and emotion regulation [36], [37]. Finally, the role of empathic concern, i.e. sympathy, in motivating prosocial behaviour has recently been studied theoretically by mathematical models [38], [39]. These models suggest that the development of empathic concern can lead to development of cooperation in economic games (termed evolutionary games by the authors). Consequently, such models suggest potential mathematical principles that could be applied in future studies to model how empathy might lead to prosocial behaviour. In parallel, our findings also suggest the potential inclusion of parameters indexing emotion regulation strategies in future models as an avenue of further research.

## Conclusion

Overall, our findings suggest that both affective and cognitive empathy are motivating factors for prosocial behaviour. However, empathy and emotion regulation can also interact to predict different levels of self-reported prosocial behaviour such that there is not always a significant positive association between affective empathy and prosocial behaviour. Our results could help to account for why associations between empathy and prosocial behaviour can sometimes be modest or weak. Our results also suggest that further investigations of the type of prosocial behaviours exhibited by individuals with varying levels of empathy and emotion regulation could be relevant as we try to understand how empathy might motivate prosocial ways of interacting with others.
